# A taxonomy of regulatory and policy matters relevant to psychedelic-assisted therapy in Australia

**DOI:** 10.1177/00048674241240597

**Published:** 2024-04-16

**Authors:** Samuel P Hatfield, Nicollette LR Thornton, Kayla Greenstien, Nick Glozier

**Affiliations:** 1 Psychological Medicine, Central Clinical School, The University of Sydney, Sydney, NSW, Australia; 2 Australian Research Council’s Centre of Excellence for Children and Families over the Life Course, Sydney, NSW, Australia; 3 School of Psychology, Faculty of Science, The University of Sydney, Sydney, NSW, Australia

**Keywords:** Psychedelic, psilocybin, methylenedioxymethamphetamine, therapy, psychotherapy, regulation, policy, Australia

## Abstract

**Objectives:**

The Australian government recently rescheduled psilocybin and 3,4-methylenedioxymethamphetamine for limited clinical uses. This change has raised various regulatory concerns and challenges for the field of psychedelic-assisted therapy. To provide clarity, we aimed to comprehensively catalogue the matters relating to psychedelic-assisted therapy that are or could be regulated.

**Methods:**

We conducted a desktop review of the literature and current regulatory sources, semi-structured interviews with professionals who had expertise in fields relating to psychedelic-assisted therapy and a framework analysis to generate a taxonomy of relevant regulatory matters. In relation to each matter, we further identified what type of regulation (if any) currently applies to that matter, any uncertainty as to how the matter should be addressed in clinical practice in the context of current regulation and whether there are conflicting views as to how the matter could or should be further regulated.

**Results:**

The taxonomy is structured into six main regulatory domains, three of which have a substantial proportion of matters with uncertainty or conflicting views: Service Establishment, Practitioner, and Treatment Delivery. Key examples of such matters include the location of services and facilities required, which professionals are eligible to become psychedelic therapists, and with what qualifications and experience. Matters in the remaining three domains, Patient Evaluation, Drug Supply and Service Oversight, appear by comparison relatively settled, with regulation either well-established or thought unnecessary.

**Conclusions:**

The taxonomy provides a roadmap for health services establishing and implementing a psychedelic-assisted therapy program, or for government and other policymakers when determining areas that may require further regulation.

On 1 July 2023, the Therapeutic Goods Administration (TGA) in Australia reclassified psilocybin and 3,4-methylenedioxymethamphetamine (MDMA) as schedule 8 substances, for the sole indications of treatment-resistant depression and post-traumatic stress disorder, respectively (TGA, 2023c). The rescheduling applies exclusively to authorised prescribers and only when delivered as part of an approved treatment protocol (TGA, 2023c). The decision comes on the back of emerging but limited clinical evidence as to the efficacy and safety of these substances (Kisely et al., 2021; Smith et al., 2022; van Amsterdam and van den Brink, 2022). Structured protocols are commonly referred to as psychedelic-assisted therapy (PAT) and generally involve a course of psychotherapeutic supports before, during and after the administration of a ‘classic’ serotonergic psychedelic (such as psilocybin, lysergic acid diethylamide [LSD] or 5-methoxy-*N,N*-dimethyltryptamine) or the empathogen MDMA. While there are notable differences in the subjective effects of psilocybin and MDMA, and the clinical protocols accompanying them, we believe the regulatory issues are sufficiently comparable to consider them together.

The rescheduling of psilocybin and MDMA makes Australia the first national jurisdiction in the 21st century to formally recognise these substances as medicines. Elsewhere, regulatory frameworks exist that functionally allow for wellness or spiritual psychedelic use, including in the United States, the Netherlands, Peru, Jamaica, Costa Rica and several other localities, as evidenced by the robust private psychedelic retreat industry (Retreat Guru, n.d.). Special access pathways also exist for medical use under narrowly defined circumstances in (at least) Canada, Switzerland and Israel (Mocanu et al., 2022). Psychedelics were also historically legal for use in psychiatric settings in several countries, including LSD in Australia until the mid-1970s under an authorised prescriber scheme (Lomax, 2017).

The 2023 Federal regulatory changes introduced a similar authorised prescriber model, with the additional oversight of a Human Research Ethics Committee (HREC) and, in some jurisdictions, additional state- or territory-based requirements. Individual psychiatrists will need to apply for Authorised Prescriber (AP) status by submitting the proposed treatment protocol to a HREC. If the application receives approval from the HREC, it will then be submitted for final approval by the TGA. The TGA has published a guidance document that provides an overview of what details are expected to be addressed in an AP application, including details of the protocol, information about the specific substance to be administered, and the measures used to address safety, efficacy and outcomes reporting (TGA, 2023a). However, the TGA has not specified any particular standards or protocols for the psychotherapeutic supports, noting this is outside of their regulatory purview (Skerritt and Langham, 2023).

PAT sits within a broad milieu of complex supply, clinical, professional, governance and financial systems that intersect with existing regulation and/or have the potential to be regulated further (Andrews and Wright, 2022). Although Australian psychiatrists appear to be cautiously in support of the introduction of PAT, many are uncertain and somewhat concerned as to how it will operate in practice (Berger and Fitzgerald, 2023; Kunstler et al., 2023). Given the breadth of potentially relevant matters it is not hard to see why.

To better appreciate these regulatory complexities, we aimed to comprehensively catalogue the matters relating to PAT that are or could be regulated. We defined ‘regulated’ broadly as any way in which the establishment and implementation of a PAT program may be standardised or constrained. We assumed regulation to include both external government regulation (i.e. legislation, rules and policy), and instruments of professional self-regulation such as standardisation documents produced by professional bodies (guidelines, codes, standards, recommendations, statements, memorandum, etc.).

## Method

### Ethics

The study was approved by the University of Sydney’s Human Research and Ethics Committee (Approval No. 2023/236).

### Study design

The study comprised two data collection and analysis phases: (1) a desktop review of the literature, to generate a list of potential regulatory matters and arrange these into a draft taxonomy; and (2) semi-structured interviews with purposively selected experts, to obtain feedback and further input into the taxonomy.

### Desktop review

A search was conducted in the Ovid MEDLINE database using the following keywords and operators: (psychedelic OR psychedelic-assisted OR hallucinogen) AND (therap* OR psychotherap* OR medic*) AND (legal OR legislat* OR regulat* OR code OR guideline OR polic* OR ethic*). A total of 226 abstracts were reviewed, and papers were selected for further analysis based on an assessment of whether there was likely to be a discussion of matters relevant to PAT regulation, policy, ethics or program design. Information was also sourced directly from relevant government websites including the TGA, the Office of Drug Control (ODC), State health departments, the US Food and Drug Administration (FDA) and from relevant professional association websites including the Royal Australian and New Zealand College of Psychiatrists (RANZCP), the Australian Psychological Society (APS), the newly formed Australian Multidisciplinary Association for Psychedelic Practitioners (AMAPP) and the US-based Multidisciplinary Association for Psychedelic Studies (MAPS). A total of 48 academic papers and 21 organisational documents were eligible.

### Data and framework

The theoretical and operational basis of our data collection and analysis was an adapted version of the framework analysis methodology (Goldsmith, 2021). The key elements of framework analysis are: (1) initial familiarisation with the data; (2) generation of a thematic framework; (3) indexing the data points; (4) charting the data according to the framework; and (5) mapping and interpretation. Framework analysis is intended to be flexible and iterative, allowing for refinement of the framework and subsequent mapping of the data according to themes and patterns that emerge, and as such is well-suited to applied policy research (Goldsmith, 2021).

We defined our data points as any matters relating to PAT that are or could be regulated. A brief survey of the literature was conducted to become familiarised with the types of matters and to construct a plausible framework of top-level domains and categories. The literature was then examined in-depth in order to index all potential regulatory matters, which were then charted within the framework. Specific information associated with each matter was also noted, such as current regulation, unresolved issues and suggestions for future regulation.

Matters were excluded if they were not sufficiently relevant or specific to PAT, e.g. regulatory requirements that apply to healthcare generally. Furthermore, we did not index matters relating only to the administration of below- or threshold-perceptual doses (‘microdosing’) or multiple medium doses administered during psychotherapy sessions (‘psycholytic therapy’), given these have not to date generated a sufficient evidence-base and are thus currently unlikely to be approved for clinical practice.

Based on the relative distribution of matters, the categories and domains themselves were refined and rearranged within the framework. The draft taxonomy was then provided to participants for the interview phase.

### Participants

To best address our research aims, it was necessary to consult professionals with experience and knowledge relevant to PAT. Given Australia’s limited pool of such professionals, purposive sampling was the most suitable recruitment method. The sample included people known professionally to the research team, authors of relevant academic papers, experts referenced in media reports and people working in PAT research and development. Participation was sought from a range of professional fields to ensure a broad spectrum of perspectives, including psychiatry, psychology, the pharmaceutical industry, research, professional bodies and government departments. A total of 30 potential participants were contacted by email and, where appropriate, a follow-up phone call. Participants self-selected by indicating their willingness to participate and provided written informed consent.

### Interviews

Participants were asked for feedback on both the framework structure and the matters themselves, including any not yet identified matters. A semi-structured interview guide was developed consisting of questions on the scope and structure of the taxonomy framework. It was not intended that participants would systematically address all aspects of the taxonomy during the interviews, but rather only areas where they had expertise. Interviews ranging between 31 and 59 minutes were conducted via video conference and audio-recorded. As the interviews were intended to elicit further matters not yet identified in the literature search, participants were encouraged to speak at length about areas of interest. Interview notes were taken both contemporaneously and during review of the recordings.

The same framework analysis methodology was applied to the interview notes. Specifically, additional matters identified were indexed and charted, along with associated information such as unresolved issues and suggestions for future regulation. Participant feedback also led to the removal or merging of some matters that had appeared in the draft taxonomy, largely because these matters were found to be inconsequential and/or not specific to PAT. A total of 102 matters appeared in the final taxonomy. Finally, based on participant feedback, the top-level domains were further amended, and the categories re-sorted, to better reflect the order in which they would likely be considered by a health service implementing PAT.

### Analysis

‘Key Notes’ were written for each of the matters, based on a synthesis of the participants’ discussions around that matter together with the associated information derived from the literature. From this process, three factors emerged that we determined could be applied to all matters (i.e. the final mapping stage of framework analysis): (1) whether the matter was subject to current regulation (being Federal/State legislation or policy, or professional guidelines); (2) whether there was uncertainty as to how a health service would address the matter in the context of current regulation; and (3) whether there were conflicting views (between participants and/or in the literature) as to how the matter should or could be further regulated. Accordingly, we mapped the data by coding the matters on these factors.

## Results

Eleven experts participated in the interviews and provided feedback on the draft taxonomy. The participant characteristics are presented in Table 1. The final taxonomy is presented in two formats. The Taxonomy Overview (Table 2) presents a one-page summary of all domains, categories and matters. The Detailed Taxonomy (Table 3) also describes the Key Notes and coding for each matter, along with a numerical identifier of which participant(s) discussed that matter.

**Table 1.** Participant characteristics.

		*n*
Total participants		11
Female		6
Male		5
Professional field(s)^a^	PAT research	6
	Psychology	5
	Psychiatry	3
	Pharmaceutical and biotechnology	3
	Professional bodies representative	2

a Some participants were in more than one professional field, such that the sum of fields is greater than the total number of participants.

**Table 2.** Taxonomy overview.

I. Service establishment	II. Practitioner	III. Drug supply	IV. Patient evaluation	V. Treatment delivery	VI. Service oversight
**(1) Facilities** • Type • Accreditation • Licence • Infrastructure • Medical equipment • Emergency care proximity **(2) Funding** • Public sector • Medicare • Private • Drug costs **(3) Program** • Design • Consumer input • Protocol flexibility • Product information • Proprietary methods • Digital therapeutics • Music licensing • Approval • Modifications	**(4) Prescriber** • Eligibility • Experience • Delegation **(5) Therapists** • Eligibility • Credentialing • Psychedelic experience • Supervision • Supervisors **(6) Training** • Requirements • Course/provider **(7) Liability** • Insurance • Scope • Contractual	**(8) Access** • Authorisation • Registered product • State restrictions **(9) Acquisition** • Product details • Locally manufactured • Direct importation • Wholesaler importation • Biological importation **(10) Control** • Storage and disposal • Dispensing **(11) Indications** • Approved indications • Off-label use • Expanded access • Experiential use **(12) Prescribing** • State authority • Dosing • Administration	**(13) Screening/limitations** • Psychiatric contraindications • Medical contraindications • Physical screen • Concomitant medication • Minimum age • Prior use **(14) Assessment** • Condition to be treated • Suitability measures • Second opinion • Onward referral **(15) Consent** • Capacity • Patient information • Expectation/bias • Specific procedures • Side effects • Psychiatric risks • Costs • Form • Enhanced process **(16) Special populations** • Support persons • Cultural safety • Socially disadvantaged • Older patients • End-of-life patients	**(17) Therapy** • Team composition • Therapist selection • Gender • Therapeutic modality • Professional boundaries • Format • Location **(18) Dosing sessions** • Required practitioners • Recording • Consent during dosing • Touch • Aborting **(19) Safety & risk mitigation** • Personnel • Monitoring • Rescue medication • Emergencies • Incident management • Psychological adverse events • Discontinuation **(20) Post treatment** • Referral • Follow-up • Ongoing psychotherapy • Group therapy • Feedback	**(21) Compliance** • Audit • Complaints • Advertising **(22) Data** • Reporting • Registry

Domains appear in capitals at the header of each column (preceded by Roman numerals); categories in bold (preceded by Arabic numerals in parentheses); matters in dot points.

**Table 3.** Detailed taxonomy.

	Category	Matter	Key notes^a^	Participants^b^	Regulation^c^	Uncertainty^d^	Contentious^e^
I.	SERVICE ESTABLISHMENT						
1	Facilities	Type	The TGA has not set any specific restrictions on what types of facilities are permitted to deliver PAT, although it suggests it should be in an ‘accredited facility, either day hospital or inpatient setting’ (TGA, 2023b). Some suggest that PAT should only be delivered in a hospital setting (e.g. Seybert et al., 2023). However, there is a general view that day clinics are as (if not more) suited, and probably more cost effectively able, to provide the treatment. It appears that, at this point in time, NSW will only authorise treatments delivered in a hospital or licenced mental health facility with overnight accommodation.	4, 9, 11	S	+	++
		Accreditation	There are no PAT-specific accreditation requirements. The TGA will likely conduct an onsite assessment of a proposed facility as part of the AP application process.	11			
		Licence	Some States may require that a facility has a certain type of licence. For example, a Private Health Facility licence will be needed in NSW, which requires overnight accommodation and that staff give consideration to the RANZCP (2023) clinical memorandum on the use of MDMA and psilocybin.	11	S		
		Infrastructure	There are currently no mandated specifications. However, certain features are important for the setting of dosing sessions that may complicate delivery in existing facilities, such as adequate floor space (for furnishings and the like), sound proofing (from external sources of sound), bathroom with private access and security features.	7, 11			
		Medical equipment	It is unclear what minimum equipment and treatment capabilities (such as for resuscitation) are needed directly onsite, and whether these need to be directly accessible from the dosing space and/or not shared with other medical services.	10, 4			
		Emergency care proximity	If PAT is delivered outside a hospital setting it is unclear what proximity the facility needs to be from an emergency care centre.	4			
2	Funding	Public sector	There is no specific public funding allocated to PAT. Given the expected cost of a PAT treatment program, funding rules that tie reimbursement to the number of patients treated may make it difficult for the public sector to deliver PAT.		F, S	+	
		Medicare	At this point there is no PAT-specific Medicare funding (noting that perhaps this might be approved in future, such as a Medicare item for the dosing session). Certain components delivered on an out-patient basis might currently be eligible for a Medicare rebate, including: (1) psychiatric consultations, including psychotherapy sessions (if delivered by a psychiatrist); (2) psychotherapy sessions delivered by an allied health professional (e.g. a psychologist) under an MHTP, if these are ‘focussed psychological strategies’ (however, these would come out of the patient’s 10 session yearly limit); (3) group therapy sessions (if incorporated into a PAT program) delivered by a clinical psychologist under an MHTP.	5, 11	F	+	
		Private	Private health insurers might fund some components of PAT delivered on an in-patient basis, and possibly psychotherapy on an out-patient basis. A funding request would need to be made to an insurer (or other third-party payer such as WorkCover or DVA) for each patient. At this point it is uncertain which insurers will provide coverage for PAT and under what conditions/limitations.	4, 11		+	
		Drug costs	Currently there is no psychedelic product subsidised through the PBS, so all prescriptions will be privately funded. As there is no psychedelic substance currently registered on the ARTG, it is unlikely that private health insurers will provide coverage.		F		
3	Program	Design	No specific standards are mandated, other than the TGA’s general expectation that the therapy program will include the three key phases (preparation, dosing and integration) and will be modelled on research protocols (TGA, 2023a). It has been noted that there is wide variability in trial protocols, particularly with psilocybin, and not all published trials have provided substantial detail about the protocol used, therefore it remains uncertain as to what protocol designs will be considered complaint. RANZCP (2023) expects the program will be designed by a psychiatrist with prior experience treating a patient with the same substance and for the same indication.	4	F, G	+	
		Consumer input	It is felt that consumer input (e.g. from a Consumer Advisory Board and/or using patient co-design for a specific population) is both appropriate and necessary for clinical implementation. However, it is unclear as to what the extent the program may deviate from clinical trial protocols based on these inputs.	1, 11			
		Protocol flexibility	While clinical trials generally follow strict scheduling standardisation, it seems generally accepted that greater flexibility will be permitted in clinical practice (e.g. timeframe for therapy, period between psychotherapy sessions and extra integration sessions). It is unclear the extent to which programs can be individualised to the patient, reflecting a tension between the benefits of personalised therapy and the need to maintain rigorous standards.	6, 8, 10, 11			
		Product information	With no psychedelic substance currently listed on the ARTG, there are currently no product information documents. If a psychedelic substance is registered, it is unclear whether the product information can or should provide details about standards for program protocols.	7, 8			
		Proprietary methods	Some PAT protocols used in research trials claim to use proprietary methods, so careful assessment may be required in relation to methods derived from clinical trial experience (and the validity of any proprietary claims evaluated).				
		Digital therapeutics	Software-based tools are being actively developed and investigated to assist in matters such as screening, customised treatment protocols, delivery of parts of the preparatory phase, dosing and re-dosing calculations and monitoring for adverse effects, which if implemented clinically may improve scalability and accessibility, but may require registration on the ARTG.	10	F		
		Music licensing	Music is usually considered an important component of dosing sessions, and a public licence is likely required to play prerecorded music to patients, although this may already be covered by an organisation’s existing licence (Australasian Performing Right Association and Australasian Mechanical Copyright Owners Society, n.d.).		F		
		Approval	The clinical protocol will need to be approved by a HREC and the TGA as part of the AP process, including details about patient selection and exclusion, consent, the setting for administration of the drug and the psychotherapy program (TGA, 2023a). In most health services, the therapy program will also need approval from the organisation’s Medical Advisory Committee.		F		
		Modifications	At this point there is no formal method for updating the protocol approved in the AP application with the TGA, so it is uncertain whether an entirely new application will be required to make substantive program modifications.		F	+	
II.	PRACTITIONER						
4	Prescriber	Eligibility	Only a psychiatrist may apply to be an AP. There are conflicting views as to whether other appropriately trained physicians should be also eligible, such as addiction specialists and general practitioners.	8	F		++
		Experience	The TGA (2023a) requires that the AP psychiatrist has had ‘appropriate experience’. RANZCP (2023) guidelines state this includes prior experience treating at least one patient with that substance for the same indication, which some have questioned as overly restrictive given the few number of psychiatrists in Australia who currently fulfil that criteria, although others have conversely suggested this is in fact probably not sufficient.	1, 8	F, G	+	++
		Delegation	The AP must directly supply the drug to the patient (TGA, 2023a). RANZCP (2023) state the AP psychiatrist has ‘overall responsibility’ during the treatment; however, the treatment may be delivered by another psychiatrist who is closely supervised. It is somewhat uncertain whether the ‘treating psychiatrist’ needs to be a direct part of the therapy team, and whether other aspects of PAT may be delegated to other medical practitioners (e.g. safety screening and monitoring during dosing sessions).	8	G	+	
5	Therapists	Eligibility	RANZCP (2023) expects that all therapists (including co-therapists/facilitators) are health professionals registered with APHRA (i.e. medical practitioners, psychologists and nurses) or an ‘equivalent governing body’. It is uncertain which other professionals fulfil these criteria; AMAPP (2023a) have specified registered social workers, psychotherapists and counsellors.	1, 5	G	+	
		Credentialing	There is no mandated system of credentialing or registration for therapists. AMAPP (2023a) has developed a system of tiered credentialing based on core training, experiential learning and dosing experience. It is unclear whether this system will be endorsed by the professional bodies or otherwise become a de facto requirement such as through the AP process.	1, 4, 5, 8			++
		Psychedelic experience	While some sources encourage therapists to obtain personal experience with psychedelics (Emmerich and Humphries, 2023) notwithstanding the lack of existing legal routes to do so, at least in Australia, others have expressed concern about this practice, e.g. due to a perception that it inflates treatment expectations (Kious et al., 2023). It is unclear whether therapists can or should disclose their psychedelic experience to patients.	1			++
		Supervision	Professional supervision at a higher frequency than normal is likely required, particularly for therapists new to PAT, given the high level of professional boundaries required and novel situations that are likely to arise.	11			
		Supervisors	There are no established requirements to become a professional supervisor for therapists conducting PAT. AMAPP (2023b) has proposed standards to become a credentialed ‘psychedelic supervisor’ including minimum training and dosing experience; however, the lack of people (particularly in Australia) who would meet the criteria means it is subject to a phase-in period.	1, 11			
6	Training	Requirements	While it is generally agreed that formal PAT training is required for both prescribers and therapists, there are no officially mandated standards. AMAPP (2023a) has specified what it considers to be core learning outcomes to become a credentialed psychedelic therapist, with a minimum of 100 hours of theory and didactic learning.	1, 7	G	+	
		Course/provider	There is currently no official training course endorsement or training provider accreditation system. It is therefore unclear to what extent there will be independent oversight of training, and if so by who.	2			
7	Liability	Insurance	Given its novel status and lack of established protocols, it is uncertain whether all aspects of PAT will be covered by professional indemnity insurance and/or will attract additional premiums.	5		+	
		Scope	Given the novel and perhaps unpredictable nature of PAT, the consent process will be key to mitigating liability. The delineation of responsibility between the prescriber and the therapist may be uncertain, given that in some circumstances it may be difficult to determine whether adverse psychological effects resulted from the substance or from the psychotherapy itself (Holoyda, 2023).	5, 9		+	
		Contractual	Liability in the private sector may become even more difficult to delineate if the therapy component is delivered by a therapist engaged as a contractor rather than an employee, and as such the roles and responsibilities would need to be very clearly defined contractually.	5		+	
III.	DRUG SUPPLY						
8	Access	Authorisation	As per the changes to the Commonwealth Poisons Standard that commenced on 1 July 2023, psilocybin and MDMA may be prescribed as an S8 substance for the approved indications by a psychiatrist with AP approval, first by applying to an NHMRC-registered HREC and then to the TGA (TGA, 2023c). The application will require details about the product to be used (ingredient, strength, dosage form, manufacturer), dosing regimen and clinical protocol, efficacy and adverse effects and clinical justification for use.	8	F		
		Registered product	No psychedelic product is currently registered on the ARTG, and there may be little incentive for companies to do so for substances that are unpatentable (McGuire et al., 2023), so PAT will for the immediate future require the use of unregistered substances. This will in most cases require additional State authority to prescribe, and it will be important to inform patients about the risks of using substances that have not been approved for safety, quality and efficacy.	2, 7, 8	F, S		
		State restrictions	While States generally automatically adopt the same scheduling as the Commonwealth Poisons Standard, it remains possible for States to depart from the standard and/or impose additional restrictions. At the date of this paper not all States provided specific guidance; practitioners will need to consult their respective State Department of Health to ensure they are aware of the current requirements.	7	S	+	
9	Acquisition	Product details	The ingredient, strength, dosage form and importer/manufacturer details must all be included in the AP application, and sufficient information should be available to demonstrate the product is GMP-compliant.	7	F		
		Local manufacture	Substances will need to be sought from manufacturer with a GMP licence and authorisation from the TGA (2023a) to produce MDMDA and psilocybin. The substances can only be manufactured for an AP or clinical trial, so the strength and dosage form will likely need to be determined in consultation prior to manufacture. Additionally, a State licence will be required to cultivate a biological psilocybin product.		F, S		
		Direct importation	An AP (or pharmacy on behalf of the prescriber) may apply to the ODC for a licence (once per year) and permit (one per consignment) to directly import the psychedelic substance, attaching the TGA’s AP approval (ODC, 2023).		F		
		Wholesaler importation	It is possible to acquire the substances via a wholesaler importer (the ‘sponsor’), however, the sponsor will need State approval to apply for an import licence from the ODC, and will need to provide the ODC with the AP’s approval before supplying the product (ODC, 2023).		F		
		Biological importation	Psilocybin-containing fungi products (including psilocybin extracted from fungi as opposed to purely synthetic psilocybin) requires an additional BICON permit from the Department of Agriculture, Fisheries and Forestry.	7	F		
10	Control	Storage and disposal	Specific storage, disposal and record-keeping requirements are determined by States, and there may be significant various between States, albeit there do not appear to be any special considerations over and above that of other S8 substances.	7	S		
		Dispensing	Depending on State requirements it may be necessary for a pharmacy to hold the substances on behalf of the prescriber (TGA, 2023a), and in a hospital setting this may require an application for the drug to be added to the hospital formulary.		S		
11	Indications	Approved indications	The approved indications specified in the Poisons Standard are treatment-resistant depression for psilocybin and PTSD for MDMA (TGA, 2023c), and the TGA (2023a) also requires that other treatments must have been trialled first. It is uncertain what thresholds apply (e.g. number of prior treatments, length of time since diagnosis).		F	+	
		Off-label use	No psychedelic product is currently registered, and in any event the substances remain S9 for any other indications, so off-label prescribing is not currently possible.		F		
		Expanded access	A SAS application can theoretically be made to prescribe psychedelics as an S9 medicine for other indications (e.g. end-of-life anxiety, chronic pain, substance use disorder, personality disorders). However, this would also require an application for State approval, which prior to rescheduling were generally denied (Watson and Kotler, 2022). Furthermore, the TGA (2023a) currently states that the SAS is ‘not open’ for psychedelics. With special access pathways now being increasingly utilised in Canada and the United Kingdom (Mocanu et al., 2022), it is unclear whether the position on SAS approvals in Australia will change in the near future.	10	F, S		
		Experiential use	There is no established access to psychedelics for non-medical purposes such as professional experiential learning. Canada has permitted access for training purposes via its Special Access Program (Mocanu et al., 2022), and it remains to be seen whether a similar exemption might someday be granted in Australia (outside of a research context).				
12	Prescribing	State authority	In some States an authority application or notification to the Department of Health may be required for each patient (e.g. NSW and Victoria), while no separate authority may be required in other States (e.g. Queensland). Practitioners will need to consult their respective State Department of Health to ensure they are aware of the current requirements.	3, 7	S		
		Dosing	Careful consideration will need to be given to dosing ranges in the AP application. It is not clear how exact doses should be calculated for individual patients, or the extent to which it is possible to titrate dose to patient response (e.g. within a dosing session or where the therapy program incorporates multiple dosing sessions).	8			
		Administration	All psychedelics must be prescribed as supervised doses, and patients cannot access the substances at any other time (TGA, 2023a).		F		
IV.	PATIENT EVALUATION						
13	Screening/ limitations	Psychiatric contraindications	Psychiatric exclusions in research trials have generally included psychosis (including family history), mania, acute suicidality, substance use disorders and violence towards others (APS, 2023; Rucker et al., 2018). The extent to which these will remain absolute contraindications in clinical practice is unclear.	2, 3, 9, 11			++
		Medical contraindications	Physical conditions excluded from research trials have included significant cardiovascular, liver, renal and neurological disease, and pregnancy (Smith and Appelbaum, 2021). The extent to which these limitations are relative is unclear.	2, 11			
		Physical screen	It is unclear whether and if so which physical screening measures should be performed routinely, e.g. blood pressure, ECG for QT prolongation (FDA, 2023).				
		Concomitant medication	Research trials have generally required discontinuation of antidepressants prior to dosing, and possibly also antipsychotics that block 5-HT2a such as quetiapine and olanzapine (Munafò et al., 2022). It is unclear whether these should be treated as absolute contraindications (e.g. relating to a theoretical risk of serotonin syndrome), or whether they are primarily relevant to efficacy.	8			
		Minimum age	At this point it seems very unlikely that PAT would be suited to paediatric/adolescent populations. It is unclear whether PAT is suitable for younger adults, given the effects on personality and decision-making processes, and what would be a suitable age threshold.	3			
		Prior use	It is unclear whether limitations should apply to patients with a history of misuse of drugs, and in particular psychedelic substances.	9			
14	Assessment	Condition to be treated	The only formal requirement is a diagnosis for an approved indication where other treatment options have failed (TGA, 2023a). State authority may impose additional requirements (e.g. NSW). There is a general assumption that this will be illness at the more severe end of the spectrum, albeit some have suggested mild–moderate illness may in fact be more suited to PAT. Other diagnostic factors are likely to be relevant, such as the severity of certain symptoms (Kelly et al., 2021), however, these are yet to be explicated.	3	F, S	+	++
		Suitability measures	Extra-diagnostic factors are also likely to be important when assessing suitability for PAT, including personal characteristics (e.g. motivation for personal change, cognition style, level of vulnerability and anxious coping styles) and extrinsic factors such as social support. It is unclear how these are to be formally assessed (e.g. through clinical judgement or by particular measures).	3, 6, 9			++
		Independent opinion	There is no specific requirement to seek an independent opinion, however, it may be appropriate to consult with the patient’s GP or existing psychiatrist.	11			
		Onward referral	Many patient applicants may be assessed as unsuitable for PAT, so standard referral pathways to other services will likely be needed.	11			
15	Consent	Capacity	A patient’s capacity needs to be assessed to ensure they can understand and realistically appraise the complex and uncertain risk/benefit profile of this novel treatment, particularly in patients who may be desperate, e.g. end-of-life patients or those with intractable illness (Rosenbaum et al., 2023). Whether this is possible given the nature and diversity of psychedelic experiences is highly debated.				++
		Patient information	All aspects of the treatment need to be explained, including the intense changes in consciousness that patients may undergo, the possibility of highly challenging experiences and very high levels of acute anxiety, and the difficulty of withdrawing from dosing once it has begun (Anderson et al., 2020).	7			
		Expectation and bias	Patients may come with unrealistic expectations (often from anecdotal or media sources) that need to be managed, and practitioner enthusiasm for the treatment is a noted further source of confirmatory bias, both of which could be iatrogenic if the patient’s experience does not meet their expectations.	10			
		Specific procedures	Consent should be sought for procedures that might be used during dosing, such as the use of therapeutic touch, and the administration of a sedative or antipsychotic in the case of a highly agitated response (Holoyda, 2023).				
		Side effects	While the side effect profile of psilocybin is generally favourable (Aday et al., 2023; Marks and Cohen, 2021), given the research is still not sufficiently developed it must be emphasised that it is not possible to rule out unusual side effects. By contrast, MDMA has a greater side effect profile that is relatively well characterised (FDA, 2023).	7			
		Psychiatric risks	Risks include the possibility of trauma re-exposure, greater suggestibility and vulnerability (including to therapist influence), flashbacks and the theoretical risk of new-onset psychosis (Smith and Sisti, 2021).				
		Costs	The substantial cost (and time) of treatment needs to be made clear, particularly in the context where efficacy both short and long term cannot be guaranteed.	8			
		Form	A detailed and bespoke consent form is likely required to capture and document these considerations.	11			
		Enhanced process	Many argue an ‘enhanced consent’ process is required that is more comprehensive than normal consent and is revised throughout the preparation phase, given that PAT has the possibility to cause major changes in personality, worldview, spiritual beliefs and life decisions (Barber and Dike, 2023). It is unclear what is an acceptable minimum process and how this is to be documented.				
16	Special populations	Support persons	Protocols may need to be adapted to enable the presence of other persons during therapy, such as translators for linguistically diverse patients and support persons for patients living with a disability. It is unclear whether a support person should be present during all or part of the dosing sessions.	1, 8			
		Cultural safety	Protocols may need to be adapted to ensure cultural safety for patients from particular religious, cultural or indigenous backgrounds (Rea and Wallace, 2021).	1			
		Socially disadvantaged	The cost of PAT will present significant barriers for socially disadvantaged patients (McGuire et al., 2023). This will likely be exacerbated by regulatory requirements that increase these costs (such as the requirement for a medical practitioner to be part of the therapy team).	1, 3, 11			
		Older patients	There is little evidence about efficacy and safety in older patients, so it is unclear to what extent a program can be designed to accommodate the risks.				
		End-of-life patients	There may be additional considerations for intake and consent when working with end-of-life patients, given they are particularly vulnerable, and given that PAT might lead to changes in decisions about irreversible matters such as euthanasia, wills and preferences for medical care (Peterson et al., 2023).				
V.	TREATMENT DELIVERY						
17	Therapy	Team composition	PAT in clinical trials is commonly delivered by a ‘therapy dyad’ consisting of a lead therapist (who is generally present at all stages) and a secondary co-therapist/facilitator. The TGA (2023a) expects that the ‘minimum standard’ for those involved in ‘patient oversight’ (presumably the lead therapist) will be a clinical psychologist, and RANZCP (2023) currently expect that at least one of the therapists will be a medical practitioner. AMAPP (2023a) has developed a detailed system for determining the dyad composition, but this differs from the RANZCP requirements in that it only requires either one of the therapists to be APHRA-registered.	1, 3, 9	F, G	+	++
		Therapist selection	Patient needs and preferences should ideally be considered when selecting the therapy team. There should be no dual relationships between the therapists themselves.	6			
		Gender	While it has been suggested that the therapy dyad should comprise one male and one female to reduce the likelihood that sexual boundaries will be transgressed (Holoyda, 2023), there is no evidence to support this as a safety measure. Furthermore, heteronormative roles might be inappropriate for some patients, e.g. some gender/sexually diverse patients or victims of sexual abuse (Rea and Wallace, 2021).	1			++
		Therapeutic modality	There is no standard modality for the psychotherapy, but the approach should be based on a model that is evidenced-based (Cavarra et al., 2022). Some suggest that a psychodynamic or DBT approach is important.	3, 6			++
		Professional boundaries	Patients receiving PAT may be particularly vulnerable to suggestion and influence, necessitating strict professional boundaries to avoid imposing (even inadvertently) the therapist’s worldview on the patient (Dupuis and Veissière, 2022; Palitsky et al., 2023). It is not clear how such safeguards should be implemented systematically.	6			
		Format	At this point it is assumed that psychotherapy will be provided on a one-on-one basis. With further research, it may be that some aspects of the therapy can be delivered in groups and/or using digital apps as a cost-saving measure.	6, 10			
		Location	There is no specific requirement for the psychotherapy sessions to occur in the same location as dosing. It is unclear whether telehealth is appropriate for some (or all) of the preparation and integration therapy sessions. In NSW it is uncertain whether the facility requirements apply only to the dosing session or also to the psychotherapy.	5	S	+	
18	Dosing sessions	Required practitioners	It has been generally expected that both members of the therapy dyad will be present during the dosing session, although note that some clinical trials are now exploring the possibility of one monitoring therapist only. If the therapist(s) present does not include the treating psychiatrist, it is uncertain whether that psychiatrist (and/or the AP) needs to be closely available during dosing.	4	G	+	
		Recording	It is often recommended that dosing sessions are video (or audio) recorded to enhance patient safety, although this will require patient consent, and may raise privacy issues. This may also place an onus on the health service to review the recording in the case of any reported anomalies, which will be a time-consuming and costly process given the length of dosing sessions.	6			++
		Consent during dosing	There may be practical difficulties relating to consent for specific procedures during dosing session. For example, confirmation of consent and/or ostensible withdrawal of consent may be ambiguous due to the patient's overwhelmed conscious state (Smith and Sisti, 2021). It is unclear how such situations should be managed and documented.				
		Touch	There are differing views as to whether it is appropriate for therapists to provide touch. It is unclear whether touch should only be patient initiated (except in the case of an emergency intervention) and what kinds of touch are ethically permissible. Current clinical trials allow for touch ranging from supportive hand holding to ‘focused bodywork’ (MAPS, 2021).	6			++
		Aborting	It is unclear what threshold should be applied for initiating an attempt to abort a psychedelic experience using rescue medication, e.g. on patient request (which may be ambiguous), extreme levels of distress or attempts to harm oneself or others.				
19	Safety & risk mitigation	Personnel	It is unclear what medically trained personnel (other than the therapists) need to be onsite or closely available during dosing, such as nurses and/or physicians.	4			
		Monitoring	It is unclear whether there should be routine monitoring of vital signs for all patients, or for a particular subset, e.g. those with known physical comorbidities or over a particular age. Further research is being conducted into the use of digital monitoring apps for monitoring purpose.	10			
		Rescue medication	It may be necessary to have ready access to sedatives and antipsychotics, and a person qualifies to administer them, should it be necessary to abort a psychedelic experience.	4			
		Medical emergencies	There should be specific processes for dealing with medical emergencies, including escalation protocols and means of accessing medical treatment not available onsite.				
		Incident management	Protocols may need to be developed for responding to non-routine circumstances that might be expected to arise, e.g. unwanted intrusion or building evacuation during dosing, or an attempt by a patient to leave the facility while still under the influence of the substance.				
		Psychological adverse events	Patients should be monitored post-dosing for adverse effects such as trauma provoked during dosing or trauma re-exposure, suicidality, flashbacks and psychosis (RANZCP, 2023).		G		
		Early discontinuation	It is unclear what strategies need to be in place for the situation where a patient withdraws from a PAT program after dosing but without having completed the integration phase.				
20	Post-treatment	Referral	Standard referral pathways should be developed for ongoing psychological support, ideally with a psychologist that the patient has an existing relationship with (or at least arranged early in the therapy program). Referrals to other community services may also be appropriate.	6, 11			
		Follow-up	A standard protocol for patient follow-up should be developed to monitor for efficacy and adverse outcomes (RANZCP, 2023). It is uncertain what are the appropriate number of and intervals between follow-ups.		G	+	
		Ongoing psychotherapy	Some suggest that there should ideally be an option to continue psychotherapy with the same therapist after the cessation of the formal integration phase, however, others suggest this raises possible boundary and/or conflict of interest issues (particularly if this was not arranged with the patient from the outset).	3, 5, 6, 11			++
		Group therapy	There is likely to be demand among patients for further integration in peer groups, though it is unclear whether this should be arranged and/or delivered by the PAT health service.	8, 11			
		Feedback	It is generally agreed that there should be a dedicated feedback process, so the program can be improved based on patient experiences.	4, 11			
VI.	SERVICE OVERSIGHT						
21	Compliance	Audit	There is currently no formal mechanism for review of compliance with approved protocols or other standards. It is unclear whether there will be any external audit or review processes (e.g. by the TGA as part of the AP process).				
		Complaints	There is no specific mechanism or body for complaints about PAT practitioners or services, and complaints are handled by the normal State health complaints bodies, AHPRA and the national boards.	9	F, S		
		Advertising	The Therapeutic Goods Act prohibits the advertising of prescription or unapproved substances to consumers. It remains uncertain whether a health service or individual practitioner can use the general term ‘psychedelic-assisted therapy’ (or equivalent) without identifying the specific substances when describing its services or providing public education materials. There is also concern relating to the potential for exaggerated claims about efficacy (Smith and Appelbaum, 2021), as has happened to some extent in the cannabis industry, and which is subject to consumer protection law.	2	F	+	
22	Data	Reporting	The TGA (2023a) requires that the AP submit 6-monthly reports on the number of patients commenced and treated, and adverse events should also be reported. Some States may also have reporting requirements for a facility licenced to provide PAT. For example, NSW requires a 6-monthly report on each patient treated.		F, S		
		Registry	There is a general consensus on the need to collect clinical treatment and outcomes data (Belouin et al., 2022). RANZCP (2023) states that psychiatrists must support and contribute data to an independent clinical registry, which will be established shortly by a university group. It remains uncertain what data will need to be collected and entered, at what longitudinal points, and the extent to which a patient is able to opt-out of from their deidentified data being used.	3, 4	G	+	

AHPRA: Australian Health Practitioner Regulation Agency; AMAPP: Australian Multidisciplinary Association for Psychedelic Practitioners; AP: Authorised Prescriber; APS: Australian Psychological Society; ARTG: Australian Register of Therapeutic Goods; DBT: dialectical behavioural therapy; DVA: Department of Veteran Affairs; ECG: electrocardiogram; GMP: Good Manufacturing Practice; HREC: Human Research Ethics Committees; MDMA: 3,4-methylenedioxymethamphetamine; MHTP: Mental Health Treatment Plan; NHMRC: National Health and Medical Research Council; ODC: Office of Drug Control; PAT: psychedelic-assisted therapy; PBS: Pharmaceutical Benefits Scheme; PTSD: post-traumatic stress disorder; RANZCP: Royal Australian and New Zealand College of Psychiatrists; TGA: Therapeutic Goods Administration.

a Provides a concise summary of issues discussed with participants and/or raised in the literature in relation to each matter.

b Indicates which participant/s (if any, identified by assigned number) identified and/or contributed to the discussion of this matter.

c Denotes current regulation (in Australia): F: Federal (Commonwealth) legislation and policy; S: State (including Territories) legislation and policy; G: professional guidelines.

d + indicates uncertainty as to how a health service and/or practitioner will address this matter under existing regulation.

e ++ indicates conflicting views (expressed by participants and/or in the literature) as to how matter could or should be regulated.

International readers should note that Australia has a federal (two-tier) system of government regulation, with Federal regulation applying across the country and State regulation applying only within that jurisdiction. In the coding of the taxonomy, (F) is used to designate that Federal regulation applies to that matter and (S) is used to designate that State regulation applies. These include both formal legislative instruments and published departmental policies and procedures that in practice direct or constrain the operations of a health service and/or practitioner. (G) refers to guidelines and other standardisation documents produced by non-government professional bodies.

The final taxonomy domains were Service Establishment, Practitioner, Drug Supply, Patient Evaluation, Treatment Delivery and Service Oversight. A description of each domain follows, together with a brief outline of what were considered the most pressing or contentious matters in each domain.

### Service establishment

This domain contained matters that would likely be prominent during establishment of a new PAT program within a health service. There were matters with relative regulatory uncertainty across almost the entire domain. A key example was the facility: although psychedelics are considered relatively safe (Evans et al., 2023; *cf.* Schlag et al., 2022), there is uncertainty and a lack of consensus as to the determination and regulation of appropriate facility types and the accessibility of emergency care in clinics operating outside of a hospital environment. The TGA noted that administration and monitoring of treatment should be in an ‘accredited facility, either *day hospital* or inpatient setting [our emphasis]’ (TGA, 2023b). However, one jurisdiction in Australia (NSW) has issued regulations requiring PAT to be provided in a mental health facility with overnight accommodation, a regulation not required elsewhere. This will have direct implications for cost and accessibility, and there were conflicting views between participants as to whether this requirement was unnecessarily restrictive or necessary from a risk perspective.

Funding presented some uncertainty, with a course of PAT expected to cost between AU$25,000 and AU$35,000 (Chrysanthos, 2023). It seems unlikely that PAT will be government-funded under current reimbursement mechanisms, and review of private health insurance reimbursement, public hospital activity-based funding and Medicare items (e.g. if the treating psychiatrist must be present for the entire treatment duration) will be required if PAT is to be at all accessible.

### Practitioner

This domain refers to the regulation of professionals who will administer and/or oversee the treatment, such as qualifications and training. Substantial uncertainties were identified, reflecting the fact that there is no established clinical framework for managing what will likely be a multidisciplinary practice. For example, the TGA guidance is nonspecific as to which professions are eligible to provide therapy. RANZCP guidelines state that therapists must be registered with the Australian Health Practitioner Regulation Agency (AHPRA) or an equivalent governing body, but unhelpfully do not specify what these other ‘governing bodies’ are. Furthermore, there are no consistently accepted standards or mechanisms for assessing what training would qualify a therapist to provide PAT, which may have implications for liability and insurance. Participants held conflicting views as to which professionals should be eligible to provide PAT, with what experience, the amount of training and ongoing professional development required and how accreditation should operate.

### Drug supply

This domain refers to mechanisms for obtaining and supplying the actual compounds. There was greater certainty in this domain, as the existing regulatory systems for Schedule 8 substance are relatively defined (albeit with some complexity). There are notable differences between States in terms of additional requirements to prescribe the substances, with some States requiring authorisation (NSW) or notification (Victoria) on a per patient basis, and other States (such as Queensland) not imposing any further requirements. However, there was no real contention among participants about these matters, as most viewed them as fixed and ultimately workable (if at times cumbersome) administrative requirements.

### Patient evaluation

This domain refers to those matters that would arise in the process of evaluating and accepting a patient for a course of PAT. This domain is relatively unregulated and, for the most part, participants did not hold strongly conflicting views about the need for further regulation. It would seem participants felt this was an area where existing clinical guidelines and professional judgement was sufficient to enable evaluation of patients for the indicated and approved conditions, albeit there was some practical uncertainty in relation to the screening of patients.

### Treatment delivery

This domain contains matters that would arise during the provision of the treatment itself. There was substantial uncertainty along with conflicting participant views in relation to matters in this domain, perhaps unsurprisingly given PAT has to date only been delivered in clinical trial settings. For instance, there is some confusion as to the allowable composition of the treating team, with the TGA (2023a) suggesting a minimum qualification of clinical psychologist for those with ‘patient oversight’, and the RANZCP (2023) guidelines requiring that at least one of the therapists is a medical practitioner. AMAPP’s (2023a) proposed composition requires at least one of the treating therapists to hold AHPRA registration (from any profession) combined with PAT-specific training. There are also several as-yet unregulated matters where conflicting views were expressed as to whether and how they should be regulated, such as whether a therapist should be able to continue providing psychotherapy to a patient following cessation of the formal integration sessions.

### Service oversight

This domain includes administrative or compliance matters where regulation imposes ongoing requirements not relating to the actual treatment of a specific patient. Participants identified some uncertainty in this domain, such as whether a health service or practitioner can use general terms like ‘psychedelic therapy’ without directly naming the substances when describing its services or providing public-facing materials. Relevantly, the Therapeutic Goods Act 1989 prohibits direct advertising of prescription-only medicines, and there have been a number of recent proceedings and convictions of medicinal cannabis providers for breach of these regulation (TGA, n.d.).

## Discussion

We developed a taxonomy of 102 matters relevant to PAT that are or could be regulated, structured into six broad domains and a number of categories. Our analysis revealed uncertainty and contention to varying degrees across the domains. We emphasise that this was not a quantitative process, so we have not made any quantitative comparisons between the domains. We also emphasise that, while the domains were intended to structure the matters in a sequence that a health service might logically follow, in practice the domains are not in fact independent, and any number of the uncertainties identified might be concurrently operating. Readers are encouraged to consult the full taxonomy for an appreciation of all matters that may be relevant to them. Nonetheless, we felt there were some particularly important or pressing matters that warrant further discussion.

Current regulation stipulates that only psychiatrists may apply for AP status, with an expectation that the AP will ‘provide ongoing psychotherapeutic management’ (TGA, 2023a) rather than simply prescribing and then delegating the psychotherapy aspects of the treatment entirely to others. The wisdom of this requirement was questioned by some participants, who pointed out that psychiatry is already undersubscribed and overburdened. They suggested that other professionals may be equally placed to perform these responsibilities, such as psychologists, or other medical practitioners like addiction medicine specialists or general practitioners with mental health credentials. Relatedly, the requirement that at least one of the therapists is a medical practitioner (RANZCP, 2023) may add substantial cost to a treatment course and so potentially contribute to further inequities in treatment access. We are not aware of any research suggesting that patients of medical practitioners have more positive outcomes relative to other mental health professionals in the psychotherapeutic management of PAT.

It was observed by some participants that, while it may seem self-evident that regulation is required to appropriately manage the risks of PAT – and to protect the public from unscrupulous operators – it is also arguable that some matters may be best left unregulated, particularly while the treatment is still largely experimental and while many questions remain to be addressed through further research. For example, while it might seem desirable to regulate some kind of minimum standards in terms of psychotherapeutic support, overregulation at this point could have the effect of stifling further exploration of therapeutic models, with the potential for undesirable consequences in terms of reduced treatment efficacy and increased cost. Some participants (who were health professionals themselves) also raised the danger of ‘overmedicalisation’ of PAT whereby, due to inflexible regulatory standardisation, the therapy becomes only available in ‘one-size-fits-all’ protocols administered by doctors ill-suited to deal with the wide range of experiences these substances elicit. Given the profound, often spiritual, frequently challenging and highly personal nature of these experiences (Kelly et al., 2021) together with their tendency to increase suggestibility and vulnerability to influence including from those who administer the therapy (Cavarra et al., 2022; Palitsky et al., 2023), the medical model itself may carry a risk of harm if these issues are not appropriately considered.

On the other hand, and perhaps unsurprisingly given the novelty of the treatment and the speed at which the scheduling changes were implemented by the TGA (Kisely, 2023), participants also identified the risks of under-regulation of certain matters. A key example is the matter of training and credentialing: while both RANZCP (2023) and APS (2023) concur that appropriate training is required for all therapists, at the time of publication there is no clear direction as to what constitutes appropriate training, nor are there regulatory systems for evaluating the growing number of PAT training programs available in Australia and online. Furthermore, neither RANZCP nor APS (nor any other preexisting body) may be well-positioned for this task, given it would need to cover practitioners from a range of professions outside their formal remit. AMAPP (2023a) has proposed that it acts as an independent cross-disciplinary credentialing body; notably, an analogous system of trans-professional credentialing is being established for practitioners who have training and experience in treating eating disorders (Hurst et al., 2022).

Several limitations to this study warrant consideration. First, it is possible that the sampling pool was overly represented by those in clinical research and drug development, which may have led to a bias that precluded the identification of other relevant matters. However, given the unexpectedly speedy down-scheduling by the TGA together with the peculiarities of implementing PAT into clinical practice, people closely involved in the current clinical research space were arguably those who could provide the most relevant data. Relatedly, a more in-depth thematic analysis might have uncovered matters not obviously apparent in the data we reviewed. However, most matters were relatively straightforward, at least in terms of identifying and defining them, and appeared across multiple sources. This suggests other approaches would have been unlikely to reveal substantial additional matters. Accordingly, we are confident the approach taken was sufficiently robust, particularly in the context of the need for a timely review in a rapidly evolving policy environment.

In addition, substantial components of the psychotherapeutic components of modern PAT clinical trials derive from so-called ‘underground’ uses in the era of prohibition, in conjunction with methods developed in the 1950s–1970s when LSD was widely researched in psychiatric use. There are likely experiences and expertise from underground and historic use that are relevant to developing contemporary systems of regulation that we did not obtain through our sampling. Notably, in Australia abuses involving LSD at the Newhaven Hospital in the late 1960s and early 1970s occurred under an authorised prescriber scheme that had the semblance of stringent oversight but lacked substantive authority (Lomax, 2017). Examining gaps in regulation and implementation in historic cases may be vital to building a safer system for patients in the contemporary era.

Future directions might include a study to systematically acquire feedback on the desirability of further regulation for those matters where there is currently uncertainty and/or conflicting views, e.g. via written surveys using numerical scales that are distributed to a wider range and number of health professionals. In addition, the taxonomy itself will likely become quickly out-of-date, given the speed at which this field is developing. Accordingly, it may be useful to periodically review and update the taxonomy.

To our knowledge, our study is the first to systematically identify and consolidate the regulatory matters relevant to PAT. The specificity of our taxonomy to the Australian context means it may be useful for Australian health services to scope and cost the establishment and ongoing delivery of a regulatory-compliant PAT program. It may be a convenient roadmap for a psychiatrist preparing an AP application and/or during the implementation phase of a program. Regulatory bodies may find use in the taxonomy as a starting point for determining whether there are any regulatory or policy gaps, and private insurers may find it useful to orient themselves to the regulatory requirements when determining the scope of coverage they may provide. Finally, professional bodies may make use of the taxonomy to review and update their guidelines.

In conclusion, we were able to identify a comprehensive set of regulatory matters relating to PAT, and structure these in a format that enables both a quick survey of the field and more detailed analyses. Crucially, the project demonstrated that there are many uncertainties, and in some cases conflicting views, as to how certain matters could or should be regulated. The taxonomy thus remains a work in progress, mirroring the field itself. Regulation may be somewhat indiscriminate, but it is one of the few tools available to ensure patient safety and, hopefully, equity in the medical use of psychedelics. The taxonomy in this paper is intended to bring the regulatory considerations and possibilities into greater focus, in the hope that this will ultimately improve outcomes for patients receiving psychedelic treatments.
